# Fast track extubation in paediatric cardiothoracic surgery in developing countries

**DOI:** 10.11604/pamj.2019.32.55.14019

**Published:** 2019-01-30

**Authors:** Federica Iezzi, Michele Di Summa, Paolo Del Sarto, James Munene

**Affiliations:** 1 Department of Paediatric and Congenital Cardiac Surgery and Cardiology, Azienda Ospedaliero, Universitaria, Ospedali Riuniti Ancona "Umberto I, GM, Lancisi, G.Salesi," Ancona, Italy; 2 Department of Cardiothoracic and Vascular Surgery, Kenyatta National Hospital and University of Nairobi, Nairobi, Kenya; 3 Department of Anesthesia and Critical Care, Heart hospital "G. Pasquinucci," Fondazione Toscana Gabriele Monasterio, Massa, Italy

**Keywords:** Congenital heart disease, fast-tracking, mechanical ventilation

## Abstract

In recent years, low-dose, short-acting anesthetic agents, which replaced the former high-dose opioid regimens, offer a faster postoperative recovery and decrease the need for mechanical ventilatory support. In this study, the aim was to determine the success rate of fast-track approach in surgical procedures for congenital heart disease. There is some evidence, mostly from retrospective analyses, that fast tracking can be beneficial. Ninety-one cases with moderate complex cardiac malformations were operated with fast-track protocol during cardiothoracic charitable missions. The essential aspects of early extubation in our cohort included: selected patients with good preoperative status, good surgical result with hemodynamic stability in low dose of inotropic drugs at the end of bypass, no active bleeding. In this setting a carefull choice and dosing of anesthetic agents, alongside a good postoperative analgesia are mandatory. The authors found that an early extubation (< 4 hours) can be both effective and safe as it reduces intubation and ventilator times without increasing post-operative complications in pediatric congenital heart disease. This study supports a wider use of fast-track extubation protocols in paediatric patients submitted for congenital cardiac surgery in developing countries.

## Introduction

In recent years, post-operative intensive care of children with congenital heart disease has placed an emphasis on earlier weaning from mechanical ventilation. Fast-tracking in cardiac surgery refers to the concept of early extubation, mobilization and hospital discharge in an effort to reduce the cost and perioperative morbidity [1]. Potential advantages of fast-tracking following surgery for congenital heart disease are: reduced ventilator associated complications, reduced requirements of sedatives, rapid patient mobilization, earlier intensive care unit discharges and decreased length of hospital stay. Fast-tracking is not restricted to anesthetic management; it is only made possible by using effective multidisciplinary patient management strategies. Early extubation after paediatric open cardiac surgery, as a part of fast-track strategy, is still not a common practice. Several recent studies have described early extubation in children undergoing surgery for simple congenital heart defects, in relatively low risk patients. Fast track extubation is meant as planned extubation either in the operating room or immediately upon arrival in the intensive care unit [2]. Settings in surgery for congenital heart disease where fast-tracking cannot be considered safe include all patients who are hemodynamically unstable, have coagulopathy or patients that do not meet generally accepted extubation criteria [3]. Shortening of both postoperative ventilation and intensive care unit length of stay are important goals to avoid complications related to mechanical ventilation, to improve patient outcome, and to reduce costs of intensive care treatment. We describe our experience of postoperative fast-tracking of children who underwent cardiac surgery, during charitable missions in developing countries, where resources and equipment were severely limited.

## Case study

During humanitarian cardiothoracic surgical missions in Kenyatta National Hospital (Nairobi, Kenya), both male and female patients aged 4 months to 18 years, undergoing elective congenital heart disease surgical procedures on cardiopulmonary bypass or off-pump, were included in the study. From December 2013 to April 2016, closed cardiac operations were performed in 19 (20.8%) patients that included: modified Blalock-Taussig shunt, pulmonary artery banding, patent ductus arteriosus closure, aortic coarctation and Glenn procedure. And 72 (79.2%) patients were done under cardiopulmonary bypass. This group included: ventricular septal defect, atrial septal defect, tetralogy of Fallot, atrioventricular septal defect. After median sternotomy and heart exposure, a dosage of heparin 300 UI/kg was given to obtain an ACT (activated clotting time) > 4 80 sec, vessels cannulation was performed and cardiopulmonary bypass was carried out under moderate hypothermia at 32ºC. Cold cristalloid St-Thomas cardioplegia was used, administered in the aorta after clamping, induction dosage of 30 ml/kg and repeated each 30 minutes with dosage of 20ml/kg. The hematocrit level during cardiopulmonary bypass was maintained higher than 24%. In all the open heart cases we used continuous ultrafiltration during cardiopulmonary bypass time. The essential aspects of early extubation in our cohort included: selected patients with good preoperative status, good surgical result with hemodynamic stability in low dose of inotropic drugs at the end of bypass, no active bleeding. In this setting a carefull choice and dosing of anesthetic agents, alongside a good post-operative analgesia are mandatory. Fast-tracking paediatric cardiac cases requires an anesthetic technique that allows safe early extubation either at the end of the procedure in the operating room, or within a few hours in the intensive care unit.

The patients were induced with intravenous midazolam 0.1 mg/kg, fentanyl 5-10 mcg/kg and pancuronium 0.1 mg/kg. Maintenance anesthesia consisted in boluses of low dose fentanyl at 1-2 mcg/kg (total dose less than 12.5 mcg/kg) along with boluses of midazolam at 0.1mg/kg at the beginning of cardiopulmonary bypass and small dose of halogenated agents (isoflurane 0.8-1 MAC, minimum alveolar concentration). Since november 2014 the anesthesia plan changed with the introduction of remifentanyl 0.15-0.25 mcg/kg/min plus propofol infusion of 2-5 mg/kg/hour as maintenance, along with midazolam at 0.1mg/kg and propofol infusion of 2-5 mg/kg/hour and small dose of alogenate agents (isoflurane 0.8-1 MAC, minimum alveolar concentration). Atracurium in a repeated dose of 0.5 mg/kg iv was introduced as muscle relaxant. Remifentanyl and propofol drip combined with short-acting anesthetic adjuvants, facilitated early awakening and tracheal extubation after surgery. The short acting opioid remifentanyl allows for a "high-dose opioid" technique without the need for prolonged mechanical ventilation. Many fast-track anesthesia protocols include remifentanil to blunt stress response and to insure adequate analgesia. For analgesia adjuvants intravenous paracetamol 15 mg/kg and ketorolac 0.5 mg/kg were administered before chest closure. In a remifentanyl based anesthesia, a bolus dose of morphine, 0.1-0.15 mg/kg, was administered at the sternum closure. At skin closure, bupivacaine 2.5 mg/kg diluted in saline was infiltrated at the wound and drain sites. If necessary opioids were reversed after stabilization of the hemodynamic status, in Intensive Care Unit. Supplementation with hypnotic drugs allows reduction of the opioid dose and enabling earlier extubation without compromising hemodynamic stability. Extubation criteria were fully awake normothermic patients (core temperature 36°C) with regular breathing and tidal volume of 5-7 ml/kg, normal pre-extubation arterial blood gas analysis on FiO_2_ of 0.4, optimal hemoglobin with no metabolic acidosis, stable hemodynamics with minimal inotropic support, minimal chest tube drainage and normal peri-operative post-repair transesophageal or epicardial echocardiography.

The patients undergoing atrial septal defect and patent ductus arteriosus closure, Glenn shunt and aortic coarctation were assessed to be extubated directly in operating theatre. The patients undergoing other procedures mentioned were assessed for extubation and extubated within four hours of arrival in intensive care unit. After transferring the patients from the operating room to the intensive care unit, with Venturi mask at 50%, the gasometry, along with other routine biochemical exams, was collected. No patient required vasoactive drugs for hemodynamic support. The median duration of mechanical ventilation was 2.85 hours. The incidence of postoperative complications, including pneumonia and atelectasis (8%), organ failure (1%) and re-intubation (3%) was significantly low. One patient presented respiratory acidosis with mild hypoxemia, with progressive recovery after 30 minutes, without the need of ventilatory support or chemical reversion of anesthetic agents. The reasons for re-intubation rate were hemodynamic instability and respiratory complication. Of the total 91 children and young adult included in the study, 36 patients (39.5%) were extubated in operating theatre, 16 (17.6%) were extubated within 1 hour of arrival in intensive care unit and 39 (42.9%) were extubated within 4 hours of arrival in intensive care unit. The Venturi mask at 50% is usually applied after extubation, with oxygen flow at 8-10 l/min. Its use is suspended after the arterial gasometry analysis, allowing the progression to nasal oxygen, using a spectacle-type nasal catheter holder and, afterwards, natural air. The patients are only discharged from the intensive care unit if their oxygenation is adequate without additional oxygen.

The intensive care unit internment ranged from 1 to 4 days and the hospital internment from 5 to 10 days. Despite the limited sanitarian resources of the hospital we tried to retrace protocols used in the advanced countries. Apart from the assisting surgeon and intensivist who already had overseas exposure to cardiac surgery, the local team members required major training of the specific pitfalls and limitations of the fast-track program. High level training of personnel is obligatory to success in this kind of programme, as avoidable mistakes could lead to fatalities [4]. The results of fast-track and delayed extubation calculated as percentages with respect to sex and weight were not clinically significant (p > 0.05). The results of the study validated the safety of fast-track extubation in paediatric congenital heart disease surgery patients at our paediatric cardiac surgery setup. Retrospective study in children following right heart bypass surgery, showed as early extubation and spontaneous ventilation resulted in decreased pulmonary vascular resistance, lower mean pulmonary artery pressure and fewer pulmonary complications such as pneumonia and atelectasis. Despite the above mentioned findings, there are widespread concerns about the safety of fast tracking and early extubation in this patient population [5].

## Discussion

Fast tracking in congenital heart surgery is not uniformly accepted, although it was introduced as early as the late 1970s. Large multicenter, controlled studies confirming potential benefits have not been performed; thus, concerns about the safety of such an approach remain. This is despite numerous reports about the feasibility of fast tracking in the pediatric cardiac surgery population [6]. Studies show that early extubation of elective cardiac surgery patients does not increase perioperative morbidity. The introduction of improved and new anesthetic agents such as modern inhalational anesthetics, short-acting opioids, hypnotics and sedatives with favorable pharmaco-dynamics and kinetic profiles are safe and make possible an anesthesia plan open toward the possibility of an early extubation and mobilization at the end of surgery. Aside from the anesthetic management, the surgical technique, cardiopulmonary bypass management and the postoperative intensive care unit care are important factors for safe fast-tracking, especially with more complex surgery. Efforts should be made to reduce the inflammatory response typically seen with long cardiopulmonary bypass times and circulatory arrest. Various ultrafiltration techniques are already being used frequently and newer developments such as low priming volume cardiopulmonary bypass circuits for infants and small children and the use of hypertonic solutions, are promising strategies to decrease blood transfusion requirements, extravascular lung water and positive fluid balance, all of which can influence the fast-tracking.

Ultrafiltration strategies in order to reduce the inflammatory response commonly seen in open heart surgery as continuous ultrafiltration during prolonged cardiopulmonary bypass or modified ultrafiltration at the end, are an important part of our fast-track management. Ultrafiltration strategies and a good surgical hemostatic control can influence the fast-tracking: a higher level of hematocrit beside a better fluid balance control, reduce the need of transfusion and improve the pulmonary function at the end of surgical procedures. Regardless of how soon the patient is extubated, pain management and sedation without respiratory depression become important considerations for all practitioners involved [7]. A mild respiratory acidosis is frequently encountered in children that were extubated early, however, this is typically well tolerated and has not led to any reported complications. On the basis of experience from the current study, all the mentioned advantages were achieved including better patient outcome and best possible resource utilization. There was an improvement in patient turn over from intensive care unit and improved ability to treat more congenital heart disease patients [8].

## Conclusion

With careful patient selection, fast-tracking can be performed in many patients undergoing surgery for congenital heart disease. In order to accomplish this safely, a multidisciplinary, coordinated approach is necessary. There is some evidence, mostly from retrospective analyses, that fast-tracking can be beneficial; however, prospective randomized studies are required to determine if fast-tracking improves outcome in children undergoing surgery for congenital heart disease. **Recommendation:** it was demonstrated that early extubation (within 4 hours) in children undergoing surgery for congenital heart disease had no negative affect on cardiac function [9]. The rate of reintubation following early extubation with modern anesthetics is low and mostly unrelated to fast-tracking. We attribute this to improved surgical techniques, cardiopulmonary bypass refinements such as minimal priming volumes, the use of ultrafiltration technique and the availability of drugs with favorable pharmacokinetics and pharmacodynamics. **Implementation:** with the development of short acting anesthetics, opioids and inhalational anesthetics with less cardiac depressive effects, early extubation after pediatric cardiothoracic operations can be safely achieved in selected patients.

## Competing interests

The authors declare no competing interest.
